# Carryover Effects of Pain Neuroscience Education on Patients with Chronic Lower Back Pain: A Systematic Review and Meta-Analysis

**DOI:** 10.3390/medicina59071268

**Published:** 2023-07-07

**Authors:** Seungwoo Shin, Hyunjoong Kim

**Affiliations:** 1 Gwangju Sang Moo Hospital, 181-7, Sangmujayu-ro, Gwangju 61948, Republic of Korea; ptssw94@gmail.com; 2 Neuromusculoskeletal Science Laboratory, 306 Jangsin-ro, Gwangju 62287, Republic of Korea

**Keywords:** chronic pain, explain pain, pain neuroscience education, low back pain

## Abstract

*Background and Objectives*: Because most individuals with chronic back pain (CLBP) have no specific cause, it is usually described as central sensitization. Pain neuroscience education (PNE) in top-down pain control may be effective against carryover effects; however, this remains unclear. In this study, the carryover effect was qualitatively and quantitatively synthesized and analyzed. *Materials and Methods*: Randomized controlled trials (RCTs) on PNE in individuals with CLBP were conducted using international databases until January 2023. Using RevMan5.4 provided by Cochrane, qualitative and quantitative analyses were performed with a risk of bias and meta-analysis, respectively. *Results*: Nine RCTs involving 1038 individuals with CLBP were included in the analysis. Four main results were identified: First, PNE had a short-term carryover effect on pain intensity (SMD = −1.55, 95% confidence interval [CI] = −2.59 to −0.50); second, PNE had a short-term carryover effect on pain catastrophizing (SMD = −2.47, 95% CI = −3.44 to −1.50); third, PNE had short- and long-term carryover effects on kinesiophobia (SMD = −3.51, 95% CI = −4.83 to −2.19); fourth, the appropriate therapeutic intensity of PNE for the pain intensity of individuals (SMD = −0.83, 95% CI = −1.60 to −0.07). *Conclusions*: PNE has a short-term carryover effect on pain intensity and pain cognition in individuals with CLBP and a long-term carryover effect on kinesiophobia.

## 1. Introduction

Chronic low back pain (CLBP), a representative of musculoskeletal disorders, refers to low back pain (LBP) that lasts for more than 12 weeks [1,2]. According to reported prevalence, one-third of the patients with LBP experience persistent disability three months after symptom onset, and these individuals are unlikely to fully recover within one year [3]. However, no specific peripheral or mechanical cause can be found in 85–90% of the cases [4].

According to the biopsychosocial model, chronic pain is mainly due to hypersensitivity of the nervous system rather than tissue-level lesions [5] and excessive nerve excitability within the central nervous system, called central sensitization, as found in most chronic pain patients [6]. This might be a result of the plasticity mechanism caused by negative emotions, anxiety, fear, and catastrophe [7], and negative attitudes toward pain and fear of recurrence previously reported to play an important role in contributing to the persistence of CLBP [8].

Guidelines for CLBP management recommend education as a key factor in managing LBP, and patient education is considered a treatment strategy for controlling and preventing chronic pain [9]. The musculoskeletal education model aims to explain the pain experience to patients in terms of tissue, normal biomechanics, and disease states, with emphasis on anatomy, biomechanics, and pathoanatomy [10,11,12]. However, this perspective is limited in its ability to explain persistent and complex pain conditions including peripheral and central sensitization, neuroplasticity, and immune and endocrine changes [13].

Given the biopsychosocial characteristics of patients with chronic pain, pain neuroscience education (PNE) aims to reduce the threat and improve the cognition of pain for patients [13,14,15,16]. Several studies have shown that PNE affects patients’ fear-avoidance beliefs and pain catastrophes, suggesting that pain can be reconceptualized through PNE [16,17,18]. Although previous studies have reported that PNE is effective in pain management compared to controls for the short term (<3 months) up to 12 months [19,20,21], there are insufficient data to show a clear benefit of PNE. A follow-up study of intensive-only PNE reported that it was not more effective in the intervention group than in the control group in improving pain and catastrophizing at 3-, 6-, and 12-month endpoints [22].

A recent systematic review and meta-analysis found that the treatment effect of PNE versus control had low short- and medium-term clinical relevance for chronic pain and disability [16]. In addition, when PNE was added to a pain management program, there was no clinically significant improvement in pain reduction in the short term [23], and it is unclear whether these effects were maintained in the medium-to-long term [24]. Therefore, in this review, qualitative and quantitative analyses were conducted by synthesizing randomized controlled trials (RCTs) of the carryover effects of PNE on pain intensity and cognition in individuals with CLBP.

## 2. Materials and Methods

### 2.1. Study Design

This systematic review and meta-analysis conducted qualitative and quantitative analyses to assess the carryover effects of PNE in patients with CLBP. This review was conducted according to the guidelines of Preferred Reporting Items for Systematic Reviews and Meta-analysis, and the preregistration of the study was registered in the International Prospective Register of Systematic Reviews (No. CRD42023393854).

### 2.2. Eligibility Criteria

The eligibility criteria for this systematic review and meta-analysis included participants (P), intervention (I), comparison (C), outcomes (O), and study design (SD) according to the key question strategy.

#### 2.2.1. Inclusion Criteria

Participants with nonspecific LBP for >3 months were included. Interventions included PNE and/OR therapeutic neuroscience education that explained pain alone or in combination with other interventions including exercise, dry needling, and manual therapy. For comparison, a group that did not include PNE was included as the control group, and all groups included conservative treatment, other educational methods, and usual care. Outcomes included pain intensity, pain catastrophism, and exercise phobia, which represent the degree of complaints of symptoms of chronic nonspecific LBP and groups were evaluated in short-term (<3 months) and long-term (6–12 months) follow-up studies. Databases were retrieved, and published RCTs were included in the study design.

#### 2.2.2. Exclusion Criteria

Studies were excluded if they consisted of participants with acute, pre/post-operative, specific pathological conditions (herniated discs, spinal stenosis). Additionally, studies not written in English or older than 10 years were excluded.

### 2.3. Search Strategy

Literature searches in this review were conducted from January 2023 onwards independently by two researchers experienced in meta-analysis. The search formula was formed by merging terms representing P, I, and SD and was searched with reference to medical subject headings.

Preidentified keywords, (randomized controlled trial) AND (pain neuroscience education) OR (therapeutic pain education) OR (pain education) OR (neuroscience education) AND (low back pain OR non-specific low back pain OR chronic low back pain OR chronic) Cumulative Index of Nursing and Allied Health Literature, Excerpta Medica Database (Embase), Medical Literature, an international electronic database containing index terms for back pain OR chronic nonspecific low back pain OR recurrent low back pain Analysis and Retrieval System Online (MEDLINE) and Physiotherapy Evidence Database (PEDro) were included. To review the missing papers, a systematic review of PNE for CLBP and an additional search were conducted using Google Scholar.

### 2.4. Data Extraction

Using a data extraction spreadsheet in Microsoft Excel (Microsoft, Redmond, Washington, DC, USA), studies retrieved from the aforementioned electronic databases were extracted and duplicate studies were excluded.

All study titles and abstracts were preliminarily screened by investigators. The titles, abstracts, and full-text contents of the selected studies were independently reviewed by two researchers. Disagreements during the final extraction process were resolved through discussions between the two researchers. All studies that did not meet the inclusion criteria were recorded. In cases of missing data, authors were conducted and asked to supply the missing data.

### 2.5. Risk of Bias Assessment

For RCTs, a seven-item risk of bias (RoB) tool developed by the Cochrane Bias Method Group was used. To evaluate the quality of the study, two researchers rated the risk of bias as low (+), high (−), and uncertain (?). The first researcher evaluated the risk of bias for each enrolled study. The results were independently reviewed by the second investigator to ensure accuracy and agreement.

### 2.6. Strategy for Data Synthesis

Data synthesis was performed using the software designed for systematic reviews provided by Cochrane (RevMan 5.4, The Cochrane Collaboration, Oxford, UK). A meta-analysis was performed when the same variables could be analyzed or when there were quantitative variables in the pre- and post-intervention tests. A meta-analysis was analyzed when at least 3 studies were included.

The effect size was analyzed using a random-effects model in which a standardized mean difference (SMD) was selected for the same variable and weights were reset considering heterogeneity among the study participants [25]. Effect size is the value used to demonstrate the strength of the intervention. Interpretation of effect size describes values as trivial (<0.2), small (≥0.2 to <0.50), moderate (≥0.50 to <0.80), or large (≥0.80) [26].

The homogeneity of the selected studies was confirmed through I^2^ and Cochrane’s chi-squared test; an I^2^ value of 75% or more was considered to indicate high heterogeneity, and an I^2^ value of less than 40% was considered to indicate low heterogeneity [27]. For publication bias, we used the funnel chart provided by RevMan5.4 [28]. We compared the short-term and long-term carryover effects of pain neuroscience education on pain intensity, kinesiophobia, pain catastrophizing, and therapeutic intensity.

## 3. Results

### 3.1. Literature Search and Characteristics of the Included Trials

**Figure 1:**
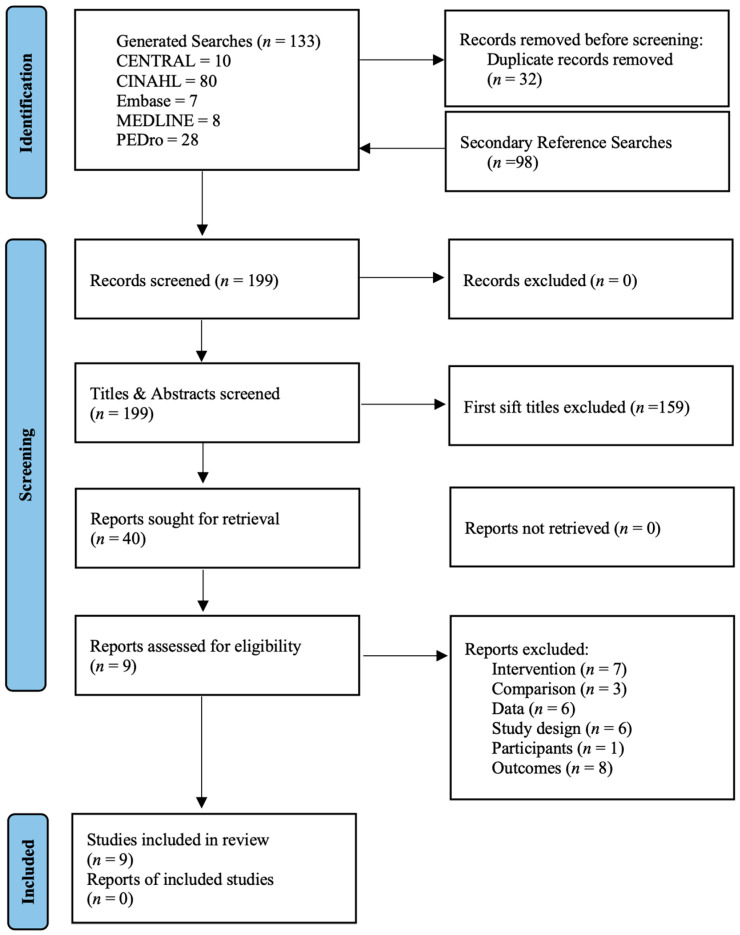
PRISMA flow diagram.

A total of 133 studies were searched in foreign databases. For missing fades, 98 papers were added using Google Scholar, and 32 papers were excluded using Excel to exclude duplicate studies. A total of 159 papers were removed after screening the titles and abstracts. Full-text reviews were removed for the following reasons: six papers for which no data were provided, seven papers with different interventions, three papers with different comparisons, six studies with different study designs, and nine papers with different participants and outcome measures. Finally, a total of nine RCTs were included in the qualitative and quantitative analyses [11,14,20,21,29,30,31,32,33] (Figure 1).

The total sample size for the nine studies was 1019 participants; PNE was conducted alone in three studies [20,21,32] and as a blended intervention with other interventions in the remaining studies. The follow-up period varied from 1 month to 12 months, depending on the papers included. To analyze the carryover effect of PNE over time, data were classified into short-term (≥3 months) and long-term effects (6–12 months). Refer to Table 1 for detailed study characteristics.

### 3.2. Methodological Risk of Bias Assessment

**Figure 2:**
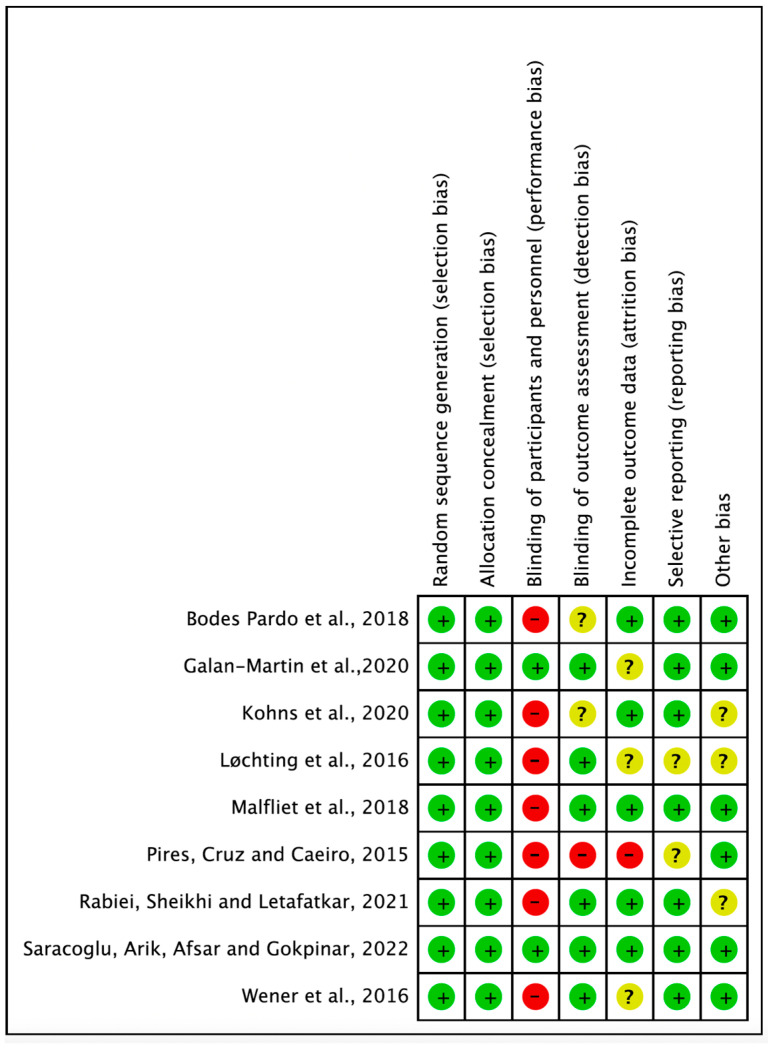
Risk of bias summary.

Methodological quality assessment of the nine RCTs showed 100% agreement among the researchers. The assessment results for the seven items of risk of bias were as follows: for random sequence generation, the risk was low in nine cases; for allocation concealment, the risk was low in eight cases and high in one case; for blinding participants and personnel, the risk was low in three cases, high in high cases, and unclear in one case; for blinding the outcome assessment, the risk was low in seven cases and unclear in two cases; for incomplete outcome data, the risk was low in five cases and unclear in four cases; for selective reporting, the risk was low in six cases and unclear in three cases; for other bias, the risk was low in four cases and unclear in five cases (Figure 2).

### 3.3. Carryover Effect of Pain Neuroscience Education for Patients with Chronic Low Back Pain

In our systematic review and meta-analysis, nine RCTs were included to evaluate the carryover effects of pain intensity, pain catastrophizing, and kinesiophobia when PNE was applied to 1019 patients with CLBP (Table 1). PNE was conducted alone in three studies [20,21,32] and as a blended intervention with other interventions in the remaining studies. The follow-up period varied from 1 month to 12 months, depending on the papers included [11,14,20,21,29,30,31,32,33].

To investigate the effect of PNE on pain intensity, a 0–10 numeric pain rating scale was used in four studies [14,21,29,31], a 100 mm visual analog scale in three studies [11,30,33], and a brief pain inventory in one study [20]. The Tampa scale of kinesiophobia was used in the selected studies to investigate the effects of PNE on kinesiophobia [11,14,29,30,31]. In addition, the pain catastrophizing scale was used to investigate the effect of PNE on pain catastrophizing [11,14,32].

### 3.4. Total Carryover Effect of Pain Neuroscience Education on Pain Intensity

**Figure 3:**
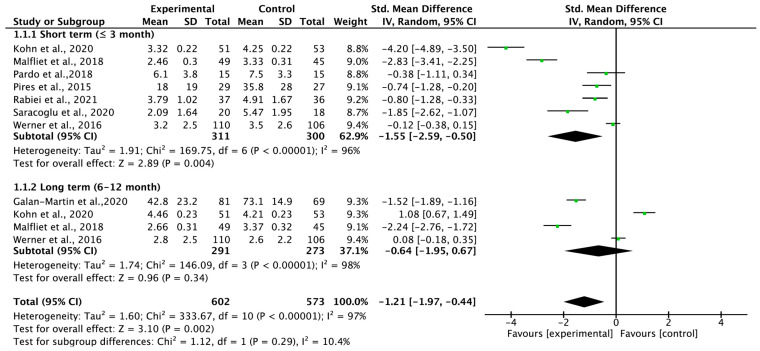
Forest plot for pain intensity.

Eight studies were quantitatively analyzed to determine the carryover effect of PNE on pain intensity according to the follow-up period (Figure 3). The carryover effect on pain intensity showed a large effect size (SMD = −1.21; 95% confidence interval [CI] = −1.97 to −0.44; heterogeneity χ^2^ = 333.67, df = 10, I^2^ = 97%; overall effect [Z = 3.10]). However, no significant effect was found in the long term but only in the short term (SMD = −1.55; 95% CI = −2.59 to −0.50; heterogeneity [χ^2^ = 169.75, df = 6, I^2^ = 96%]; overall effect [Z = 2.89]).

### 3.5. Total Carryover Effect of Pain Neuroscience Education on Pain Catastrophizing

**Figure 4:**
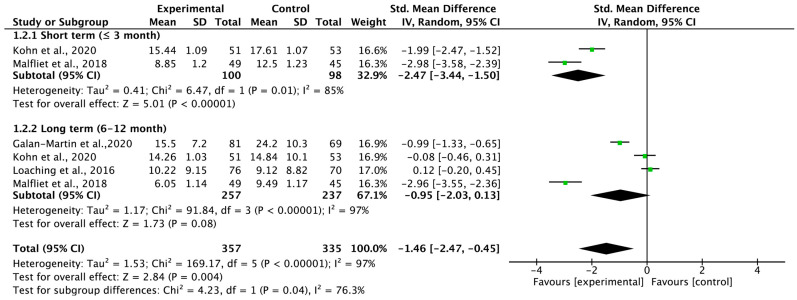
Forest plot for pain catastrophizing.

Four studies were quantitatively analyzed to determine the carryover effect of PNE on pain catastrophizing according to the follow-up period (Figure 4). The carryover effect for pain catastrophizing showed a large effect size (SMD = −1.46; 95% CI = −2.47 to −0.45; heterogeneity [χ^2^ = 169.17, df = 5, I^2^ = 97%]; overall effect [Z = 2.84]). However, no significant effect was found in the long term but only in the short term (SMD = −2.47; 95% CI = −3.44 to −1.50; heterogeneity [χ^2^ = 6.47, df = 1, I^2^ = 85%]; overall effect [Z = 5.01]).

### 3.6. Total Carryover Effect of Pain Neuroscience Education on Kinesiophobia

**Figure 5:**
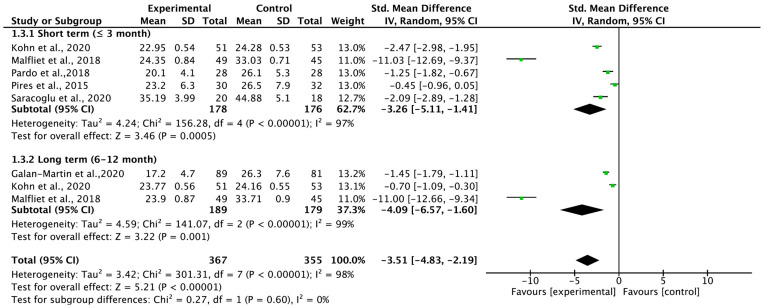
Forest plot for kinesiophobia.

Six studies were quantitatively analyzed to determine the carryover effect of PNE on kinesiophobia according to the follow-up period (Figure 5). The carryover effect for kinesiophobia showed a large effect size (SMD = −3.51; 95% CI = −4.83, −2.19; heterogeneity [χ^2^ = 301.31, df = 7, I^2^ = 98%]; an overall effect [Z = 5.21]). The results of the subgroup analysis also showed large effect sizes in both the short and long terms.

### 3.7. Total Effect of Pain Neuroscience Education on Pain Intensity According to Therapeutic Intensity

**Figure 6:**
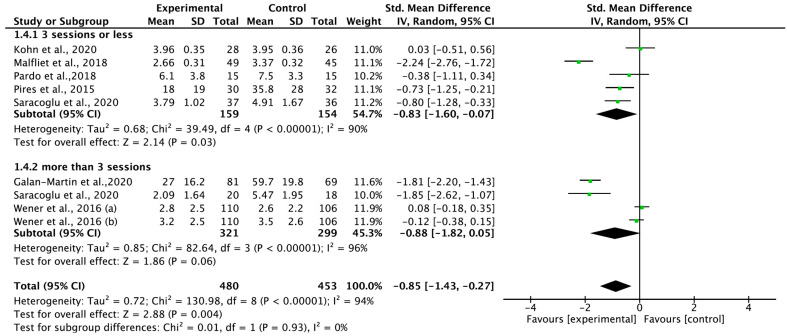
Forest plot for therapeutic intensity. Werner et al., 2016 [21] (a): long term (12 month), Werner et al., 2016 (b): short term (3 month).

Regarding the effect of PNE on pain intensity, seven papers were classified according to therapeutic intensity (less or more than three sessions), regardless of the follow-up period (Figure 6). Five papers were classified as three sessions or less, Four papers were classified as more than three sessions. PNE for pain intensity showed a large effect size (SMD = −0.85; 95% CI = −1.43 to −0.27; heterogeneity [χ^2^ = 130.98, df = 8, I^2^ = 94%]; overall effect [Z = 2.88]). In the enrolled trials, it was mainly less or more than three sessions, and a large effect size appeared only when it was less than three sessions (SMD = −0.83; 95% CI = −1.60 to −0.07; heterogeneity [χ^2^ = 39.49, df = 4, I^2^ = 90%]; overall effect [Z = 2.14]).

### 3.8. Publication Bias

Nine studies were synthesized for the systematic review and meta-analysis according to the eligibility criteria. The Cochrane guidelines recommend that publication bias is not appropriate when there are fewer than 10 synthesized studies; therefore, these were not analyzed [34].

## 4. Discussion

This systematic review and meta-analysis comprehensively, qualitatively, and quantitatively analyzed the carryover effects of PNE on pain intensity and cognition (pain catastrophizing and kinesiophobia) in individuals with CLBP using RCTs. As far as we know, it is agreed that the carryover effect is improved by blending PNE with existing interventions, so this review is the first meta-analysis to synthesize and analyze them.

In a study explaining pain, patients reported decreased brain activity in the cortical areas related to pain processing after education [35]. These results raise questions regarding the effect of PNE on the efficacy of endogenous pain suppression. Several studies have reported that the pain suppression system is not optimally assembled in patients with chronic pain [36,37]. However, changes in the pain suppression system were observed three months after pain education [15]. Thus, sufficient time is required for changes in the neural matrix in patients experiencing chronic musculoskeletal pain and central sensitization. Therefore, to investigate the effect of PNE on top-down pain processing in the brain, it is necessary to synthesize the carryover effect through sufficient follow-up.

In this review, nine enrolled RCTs, involving 1038 individuals with CLBP, were synthesized and analyzed [11,14,20,21,29,30,31,32,33]. Our results based on the effect size are as follows: First, the carryover effect of PNE on pain intensity and pain cognition in individuals with CLBP has a large effect size; second, the short-term carryover effect of PNE on pain intensity and pain cognition in individuals with CLBP has a large effect size; third, the effect size of the carryover effect on kinesiophobia in individuals with CLBP is large in the short and long term; fourth, the appropriate number of sessions of PNE for the pain intensity of individuals with CLBP is three sessions or less; fifth, giving PNE blended with interventions other than PNE alone to individuals with CLBP positively improves pain intensity and pain cognition.

Studies have utilized diagrams, pictures, and videos to reconceptualize patients’ negative beliefs about pain through PNE, which includes biopsychosocial factors [14,20,33]. This is an important process that facilitates a patient’s ability to cope with their condition. The benefits of PNE through this are supported by previous systematic reviews reporting strong evidence that explaining to patients reduces pain levels, fear avoidance, and pain catastrophizing [13,17]. However, this differs from our meta-analysis results including differences in methodological approaches and is somewhat consistent with studies in a more recent review suggesting that PNE improved pain and disability in the short term but had problems with long-term efficacy [38]. In addition, the finding that pain may not be a viable intervention to improve pain and disability is partially consistent with the findings of this study [39]. Thus, it emphasizes the importance of viewing PNE only as part of a complex biopsychosocial approach and not as an intervention for simple pain control [40].

In addition, a meta-analysis similar to this study was consistent with the results that PNE brought about clinical improvements in patients’ kinesiophobia and pain catastrophizing [16]. However, in our study, a long-term carryover effect on pain catastrophizing was not confirmed. Kinesiophobia was identified in the short and long term, which is partially consistent with the results of previous studies showing that PNE leads to particularly significant improvements in kinesiophobia [16,17,29]. However, all three studies included in the long-term carryover effect analysis showed a high degree of heterogeneity (I^2^ = 99%). These results should be interpreted with caution, and the high heterogeneity may depend on the variety of interventions, individual differences among participants, and the mode and frequency of delivery of education.

The therapeutic intensity of the PNE was further analyzed. There was a significant improvement compared with the control group in fewer than three sessions, but there was no significant difference in interpretation through SMD. It can be assumed that the tentative meaning will improve as the therapeutic intensity increases; however, according to reports from related studies, the maximum effect of PNE is achieved in individual sessions [41]. In addition, studies using one or two sessions of 30 to 45 min are increasing [41,42], which means that PNE can be easily implemented in clinical practice in such a short session and is considered to be more effective when combined with other therapeutic options.

In this review, we summarized the following points. The clinical practice of PNE, as part of a therapeutic option in a biopsychosocial approach, results in positive improvements in pain intensity and cognition in individuals with CLBP. Rather than increasing the therapeutic intensity, PNE should consider the diversity of blended interventions, individual differences among participants, educator capacity, and delivery methods, which could affect the treatment effect.

However, there were some limitations when analyzing the carryover effect of PNE in individuals with CLBP. First, PNE could have a great influence on the results depending on the competency of the educating physical therapist; second, high heterogeneity (≥ 75%) was found in the results of quantitative analysis, so generalization was limited; third, therapeutic intensity for pain intensity was classified, but other variables were not synthesized; fourth, based on the result that it could vary depending on the therapeutic intensity, it could be predicted that the carryover effect might vary depending on the therapeutic intensity. Finally, the number of synthesized studies is small, so we should be concerned about over-interpretation. Further studies require protocols for the therapeutic intensity of pain neuroscience education, guidelines for educators, and a comparison of more beneficial blended interventions.

## 5. Conclusions

We performed qualitative and quantitative analyses to provide clinical suggestions regarding the carryover effects of PNE in the management of patients with CLBP. In conclusion, PNE has a short-term (less than three months) carryover effect on pain intensity and pain cognition in individuals with CLBP and a long-term (6–12 months) carryover effect on kinesiophobia. The recommended therapeutic intensity was considered appropriate for three or fewer sessions. However, the high heterogeneity limits generalization.

## Figures and Tables

**Table 1:** Characteristics of the included trials.

Study	Participants (Sample Size)	Therapeutic Intensity	Outcome Measure	Author’s Conclusions
Galan-Martin et al., 2020 [11]	Non-specific chronic spinal pain (166)	Total intervention period = 6 weeks EG = 6 sessions of PNE (10 h), 18 sessions exercise (18 h, three sessions per week) CG = 15 sessions (15 h) usual physiotherapy treatment	Pain intensity = VAS Pain catastrophizing = PCS Kinesiophobia = TSK	PNE and PE-based playful, dual-tasking, and socialization-promoting components are most effective in improving quality of life improvement, reduction of pain, catastrophism, kinesiophobia, CS, and disability.
Kohns et al., 2020 [20]	Low back pain (104)	Total intervention period = A single PPN session EG = A single PPN session lasting 20–25 min, used a 3 min instructional video CG = Single session of self-assessment of health-related behaviors, lasting 20–25 min, used an educational video.	Pain intensity = BPI	PPN helps patients learn about centralized pain and evaluate their risk factors for such pain. Moreover, this intervention resulted in some reduction in pain intensity in the short term, but not in the long term.
Løchting et al., 2016 [32]	Low back pain (203)	EG = 30 min, one-on-one PNE once a week for 4 consecutive weeks CG = 30 min, one-on-one sessions of usual care once a week for 4 consecutive weeks	Pain catastrophizing = PCS	The Cognitive patient education programs did not lead to improvements in individuals’ quality of life and pain catastrophizing. Through cognitive interventions need to be researched further in order to strengthen the understanding of these constructs in LBP.
Malfliet et al., 2018 [14]	Chronic spinal pain (120)	Total intervention period = 12 weeks EG = 3 sessions of PNE, 15 exercise sessions CG = 3 sessions of Traditional education, 15 exercise sessions	Pain intensity = NPRS Kinesiophobia = TSK Pain catastrophizing = PCS	Combining PNE with CTMCT can reduce pain and disability and improve mental and physical functioning and pain cognitions in people with nCSP.
Bodes Pardo et al., 2018 [29]	Chronic low back pain (56)	Total intervention period = 3 months EG = 2 sessions of PNPE (30 to 50 min), TE (daily) CG = TE (daily)	Pain intensity = NPRS Kinesiophobia = TSK	Combining PNE with TE is more effective in reducing pain, disability, and pain catastrophizing for participants with CLBP, with a large effect size, compared with TE alone.
Pires, Cruz and Caeiro, 2015 [30]	Chronic low back pain (62)	Total intervention period = 6 weeks EG = 2 Group sessions, 90 min each Aquatic program: 6 weeks, 2 session/week CG = 6 weeks program consisting of 12 session of aquatic exercise (30–50 min)	Pain intensity = VAS Kinesiophobia = TSK	PNE is a clinically effective addition to aquatic exercise. Further studies are necessary to better understand how pain neurophysiology education influences pain intensity and disability and to evaluate the long terms effects of this intervention on pain and disability.
Rabiei, Sheikhi and Letafatkar, 2021 [33]	Chronic low back pain (73)	Total intervention period = twice weekly for 8 weeks EG = 3 educational sessions PNE, each lasting 30–60 min; MCE, 2 sessions a week for 8 weeks. CG = Group-based exercise (GE) program. Proposed sessions 2 times a week for 8 weeks, each session lasting 60 min.	Pain intensity = VAS Kinesiophobia = FABQ	Individual treatment involving PNE plus MCE seem to be better at reducing pain intensity and disability compared to GE, while no significant differences were observed for fear-avoidance beliefs and self-efficacy between the 2 groups in patients with CLBP.
Saracoglu, Arik, Afsar and Gokpinar, 2022 [31]	Chronic low back pain (38)	Total intervention period = 4 weeks EG = MT (2 day/week, 30 min), PNE (each week, 40–45 min), HEP (3 day/week, 10 repititions) CG = MT (2 day/week, 30 min), HEP (3 day/week, 10 repetitions)	Pain intensity = NPRS Kinesiophobia = TSK	When compared to MT and HEP or HEP alone, the combination of PNE, MT, and HEP is associated with greater improvement in terms of pain intensity and kinesiophobia in the short (4 weeks) and midterm (12 weeks).
Werner et al., 2016 [21]	Non-specific low back pain (216)	EG = 30 min. of one-to-one PNE sessions once a week for four consecutive weeks CG = 30 min. of one-to-one sessions of usual care once a week for four consecutive weeks	Pain intensity = NPRS	The equal improvement observed in both groups in our study suggests that patient education may be useful, but no clinical or health economic benefits as a result of adding a cognitive education program to usual treatment for patients with subacute and chronic LBP.

AE, aquatic exercise; BNE, back and neck education; BP, brief pain inventory; CG, control group; CLBP, chronic low back pain; CMCT, cognition-targeted motor control training; CS, central sensitization; CSP, chronic spinal pain; CTMCT, cognition-targeted motor control training; EG, experimental group; EX, exercise; FABQ, fear avoidance beliefs questionnaire; HBC, health behavior control; HEP, home exercise program; LBP, low back pain; MCE, motor control exercise; MT, manual therapy; NPRS, numeric pain rating scale; PC, primary care; PCE, pain-contingent exercise, PCS, pain catastrophizing scale; PE, physical exercise; PNE, pain neuroscience education; PNPE, pain neurophysiology education; PPN, pain psychology and neuroscience; TCE, time-contingent exercise; TE, therapeutic exercise; TSK, tampa scale of kinesiophobia; VAS, visual analog scale.

## Data Availability

Not applicable.
